# Tomography gives a new dimension to an ancient organelle

**DOI:** 10.7554/eLife.02589

**Published:** 2014-03-25

**Authors:** Malan Silva, Maureen M Barr

**Affiliations:** 1 **Malan Silva** is in the Department of Genetics, Rutgers University, Piscataway, United States; 2 **Maureen M Barr** is in the Department of Genetics, Rutgers University, Piscataway, United Statesbarr@dls.rutgers.edu

**Keywords:** cilia, electron microscopy, electron tomography, *C. elegans*

## Abstract

Advances in sample preparation and electron microscopy have allowed the structure of cilia to be explored at an unprecedented level of detail.

**Related research article** Doroquez DB, Berciu C, Anderson JR, Sengupta P, Nicastro D. 2014. A high-resolution morphological and ultrastructural map of anterior sensory cilia and glia in *Caenorhabditis elegans*. *eLife* **3**:e01948. doi: 10.7554/eLife.01948

**Image** The cilia, neurons and associated cells within the head of a microscopic worm

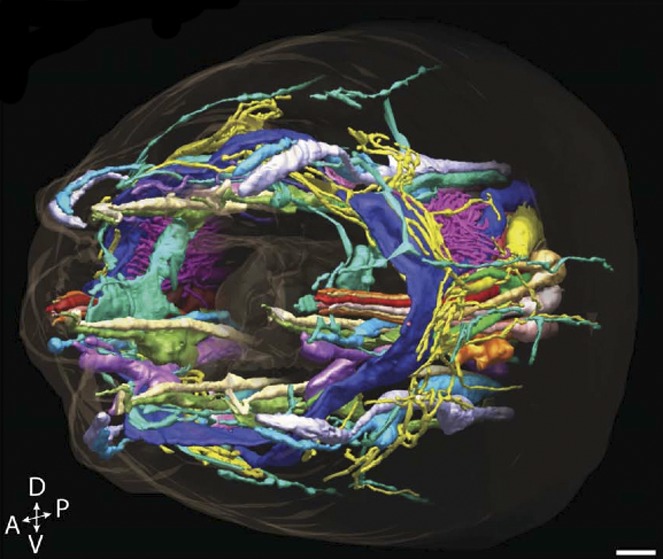

When Antoni van Leeuwenhoek—who is often considered to be the ‘Father of Microbiology’—looked down his microscope in 1675, he saw that some single-celled microbes had hair-like projections that moved with a sweeping motion. These organelles later came to be known as cilia, and they have been studied by countless biologists ever since (Beales and Jackson, 2012). These investigations have revealed that cilia can have a range of different structures and perform many different functions. We now know how a cell builds an individual cilium, but we still do not fully understand the range of cilia structures and functions observed in nature. Since the disruption of cilia can cause kidney disease, male infertility, neuropsychiatric disorders and various other ‘ciliopathies’, there are strong motivations to understand how this diversity of structures and functions arises.

In addition to the ‘sweeping’ cilia that were observed first, many cells have cilia that do not move. On neurons, these non-motile cilia are involved in detecting touch, specific chemicals, or changes in temperature. The nervous system of the worm *Caenorhabditis elegans* has been an important model for both functional and developmental neurobiology since the late 1960s (Ankeny, 2001), and a number of important breakthroughs were made in the mid-1980s. In particular, the ‘wiring diagram’ for the 302 neurons in *C. elegans* was elucidated (White et al., 1986), and the details of the sensory systems in the head and tail were worked out (Perkins et al., 1986).

Now, in *eLife*, a collaboration led by Piali Sengupta and Daniela Nicastro of Brandeis University—including David Doroquez and Cristina Berciu of Brandeis as joint first authors, and James Anderson of the University of Utah—has generated a three-dimensional reconstruction of the different ciliated-neurons and associated support cells from the head of *C. elegans* in unprecedented detail (Doroquez et al., 2014). By using the latest methods in tissue preservation and high-resolution electron tomography, Doroquez, Berciu et al. have, in effect, been able to transform old-fashioned black and white ‘snapshots’ into breathtaking 3D-models and movies.

Rapidly freezing the worms in a high-pressure environment immobilized the sample within a fraction of a millisecond and minimized shifts in the components of each cell. The worms were then cut into extremely thin slices, and electron tomography was used to create 3D models of the cells in the worm’s head from 2D electron microscopy images of each slice (for a review, see Lucic et al., 2005).

Although these techniques have already been used to examine the internal structures of cilia, this is the first time that they have been used to study a range of different types of cilia in an intact sensory system. Not surprisingly, this unprecedented level of detail revealed some features that had not been seen before (Figure 1). For example, *C. elegans* cilia were shown to lack the so-called ‘transition fibers’ that connect the cilium and the cilia base; this means that structures that were previously interpreted as transition fibers were in fact imaging artifacts. Doroquez, Berciu et al. also observed vesicles—small membrane-bound packages that move molecules around within cells—within the ciliary base or transition zone. In the past it had been assumed that cilia did not contain vesicles, so their presence suggests that an alternative means of transporting molecules might operate within a cilium.

**Figure 1.**
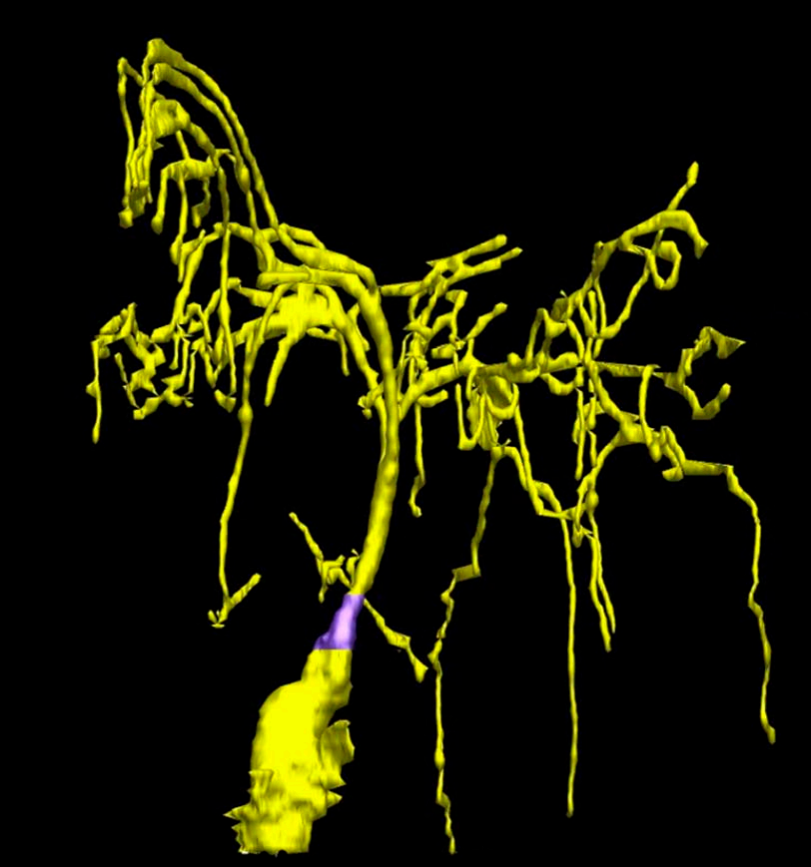
Taking a closer look at the ciliated-neurons in the head of *C. elegans*. Doroquez, Berciu et al. provide a remarkable view of elaborate sensory neurons, such as the complex branched ciliated-endings of the so-called ‘amphid wing’ neurons (yellow) that are required for detecting chemicals in the environment. These tree-like branches contain molecular filaments (called microtubules) that are arranged in a variety of different ways that had not previously been recognized. It is thought that these branches increase the surface area of the cilium, allowing the animal to better sample its chemical environment. The base of the cilium is called the transition zone (purple).

Cilia are supported from within by a molecular scaffold that is made of hollow filaments called microtubules. Previous studies revealed the presence of ‘electron-dense material’, largely proteins, inside the microtubules of the long cilia—sometimes called flagella—that are found in single-celled microorganisms and sea urchin sperm (Heuser et al., 2009; Pigino et al., 2012). Some cilia from *C. elegans* also had electron-dense material inside certain microtubules that are linked together via thin fibers. These *C. elegans* cilia are important for worm foraging and for avoiding danger, and they resemble touch-sensitive receptors called campaniform mechanoreceptors that are found in insects. This observation suggests a correlation between these specific adaptations in the shape of cilia and the cilia being particularly sensitive to touch.

The high-resolution reconstruction of the sensory system in the head of *C. elegans* reveals previously unknown and unforeseen associations between ciliated-sensory neurons and their supporting cells, called glia. Cilia and the branches of neurons, called dendritic spines, have been proposed to be comparable in their ability to convey environmental signals and nerve impulses, respectively (Nechipurenko et al., 2013). This raises an interesting question: Do cilia-associated glia function in a similar way to those at the junctions between neurons, and do they modulate a cilium’s ability to receive and/or transmit a signal?

The work of Doroquez, Berciu et al. also raises other questions. How are the elaborate ciliated-endings that are observed in the images actually sculpted? What is the biological or functional relevance of the physical connection between neuronal cilia and glial support cells? A challenge going forward is to link this reconstruction with advances in imaging. This could include tracking changes in the concentration of ions, such as calcium, that are involved in nerve impulses in living animals. Alternatively, super-resolution microscopy could be used to visualize structures of biomolecules in order to understand how sensory information is received and processed by the nervous system. This work sets the stage to address these questions, and to apply what has been learned in the *C. elegans* paradigm to more complex sensory systems and to ciliated cells hidden deep in the mammalian body, such as in the brain.
